# AD genetic risk factors and tau spreading

**DOI:** 10.3389/fnagi.2015.00099

**Published:** 2015-05-21

**Authors:** Jesús Avila, Alberto Gómez-Ramos, Marta Bolós

**Affiliations:** ^1^Centro de Investigación Biomédica en Red de Enfermedades NeurodegenerativasMadrid, Spain; ^2^Centro de Biología Molecular “Severo Ochoa” CSIC-UAMMadrid, Spain

**Keywords:** endocytosis, tau proteins, genetic factors, PICALM, BIN1 bridging Integrator 1/amphiphysin-2 gene

Development of tau pathology is associated with progressive neuronal loss and cognitive decline. In the brains of Alzheimer's disease (AD) patients, tau pathology propagates according to an anatomically defined pattern with relatively uniform distribution, and contributes to cognitive decline in age-associated tauopathy (Braak and Braak, 1991; Saito et al., 2004). Recently, it has been revealed that tau, which is an intracellular protein, can appear in the extracellular space, likely due to an exocytosis mechanism. Such extracellular tau could then be internalized into neighboring cells in at least two different ways depending on its aggregation state. In the case of soluble monomeric or small oligomeric tau protein, the endocytosis appears to be clathrin dependent (reviewed in Rubinsztein, 2006). In contrast, larger aggregates of tau could bind heparin in the extracellular matrix and be internalized through macropinocytosis (Holmes et al., 2014). As a result of exocytosis and endocytosis, the spreading of tau can occur in various neurodegenerative diseases (tauopathies) including AD. In this opinion article we have focused on the endocytosis mechanism.

Several genetic risk factors have been associated with a higher probability of developing sporadic Alzheimer's disease (SAD). The Alzheimer Association (http://www.alzforum.org/) has ranked the top six risk genes, shown in Table 1, based on genome-wide association studies (GWAS).

## Binding of SAD genetic risk factors to tau protein

The myc box-dependent-interacting protein 1, also known as bridging integrator 1 (BIN1), clusterin (clu), phosphatidylinositol binding clathrin assembly protein (PICALM), and apolipoprotein E (ApoE) are encoded by the BIN1, CLU, PICALM, and ApoE genes, respectively. These proteins are involved in the endocytosis of tau in either a direct (BIN1, CLU, and PICALM) or an indirect (ApoE) way, and all of them can also interact directly with tau (Chapuis et al., 2013; Tan et al., 2013; Holler et al., 2014; Moreau et al., 2014; Zhou et al., 2014). ApoE - mainly isoform ApoE3 - can bind efficiently to tau protein (Strittmatter et al., 1994). For BIN1, a novel brain-specific allele containing a 3 bp insertion has been reported that may be responsible for the interaction of BIN1 with tau (Tan et al., 2013). Intracellular clusterin interacts with brain isoforms of BIN1 and with tau (Zhou et al., 2014). Less is known about the interaction of PICALM and tau. However, Moreau et al. (2014) carried out groundbreaking work describing the relationship between PICALM and tau. They showed how PICALM-dependent autophagy can modulate tau accumulation in cells. Impaired autophagy could result in neurotoxicity and, consequently, might also be related to the spreading of tau pathology.

**Table 1:** **The top six AD risk genes that interact with tau**.

**Order**	**Gene**	**Location (GRCh38.p2 assembly)**	**Polymorphism**	**References**
1	ApoE	chr19:44905754-44909393	ApoE 2,3,4	Strittmatter et al., 1994; Grupe et al., 2007
2	BIN1	chr2:127048027-127107355	rs 744373	Schellenberg and Montine, 2012
3	CLU	chr:8: 27596917-27615031	rs 11136000	Lambert et al., 2009; Harold et al., 2009
4	ABCA7	chr19:1040101-1065572	rs 3764650	Hollingworth et al., 2011
5	CR1	chr1:207496147-207640647	rs 3818361	Lambert et al., 2009
6	PICALM	chr11:85957684-86069882	rs 3851179	Harold et al., 2009

ApoE and clu (ApoJ) are related proteins. They are involved in cholesterol and lipid transport and can regulate Aβ endocytosis and Aβ clearance (Bertrand et al., 1995; Nuutinen et al., 2009). They also share some cellular receptors (Leeb et al., 2014). For example, both ApoE and clusterin bind to heparin (Cardin et al., 1986; Pankhurst et al., 1998), which in turn may affect endocytic processes such as macropinocytosis. Thus, it can be hypothesized that ApoE (and ApoJ) may, in this indirect way, regulate tau endocytosis.

BIN1 has been ranked as the second most important susceptibility locus for developing SAD. It is expressed from a single locus located on human chromosome 2 (Ren et al., 2006). The gene is transcribed into nuclear RNA that can produce different proteins by alternative splicing (Pineda-Lucena et al., 2005). Some of the BIN1 isoforms, such as isoforms 1-6, are specifically located in the brain (Butler et al., 1997; Tsutsui et al., 1997; Wechsler-Reya et al., 1997). Furthermore, BIN1 is mainly expressed in neurons and some brain isoforms are mainly expressed in the axon initial segment (Holler et al., 2014).

## Extracellular tau endocytosis

Extracellular soluble tau (monomers, small oligomers) or larger aggregates of tau can be endocytosed by neurons in several ways. It was shown that neurons have cell receptors for extracellular tau, for example M1 and M3 muscarinic receptors (Gomez-Ramos et al., 2006, 2008). Once extracellular tau is bound to muscarinic receptors, it can be endocytosed in a clathrin-dependent process. This uptake mechanism could facilitate the spreading of tau from neuron to neuron, perhaps through synaptic transmission (De Calignon et al., 2012; Liu et al., 2012; Pooler et al., 2013). For aggregated tau, endocytosis is mediated by macropinocytosis (Holmes et al., 2014), in which components of the extracellular matrix, such as heparin sulfate, seem to play a role. Thus, two clearly different endocytic pathways have been proposed for the neuronal uptake of tau: receptor-clathrin dependent uptake of soluble tau species versus cell matrix-dependent endocytosis of tau aggregates.

In SAD, an increase in the expression of BIN1 and PICALM has been described, which could induce an increase in tau clathrin-mediated endocytosis. This could lead to the accumulation of soluble tau in tau recipient cells and may result in a toxic effect. On the other hand, a decrease in the level of clusterin, which like ApoE, is a heparin-binding protein, could enhance the binding of aggregated tau to the extracellular matrix and hence also enhance its endocytosis, resulting in a toxic effect in the recipient cell.

In summary, it is remarkable that four of the main genetic risk factors for Alzheimer's disease are tau-binding partners. Identifying how these risk factors affect tau propagation may unveil new therapeutic targets to stop or delay progression of pathology. For example, one possible way to reduce tau endocytosis and subsequent spreading of tau pathology may be to decrease neuronal levels of BIN1 and PICALM (which are increased in Alzheimer's disease) in early stages of the disease. Future research is therefore needed to better understand the interactions of tau with these proteins.

### Conflict of interest statement

The authors declare that the research was conducted in the absence of any commercial or financial relationships that could be construed as a potential conflict of interest.
